# Supplementary material for: A basophil-specific GPCR mediates the immune response to helminth infection
Source: bioRxiv. 2026 Apr 8:2026.04.06.716327. Preprint. [Version 1] doi: 10.64898/2026.04.06.716327 (PMC13081893; doi:10.64898/2026.04.06.716327)
Supplement: Supplement 1 [file media-1.pdf]

Supplementary Materials for

A basophil-specific GPCR mediates the immune response to helminth infection

Aleksander Geske *et al.*

*Corresponding author. Email: Xintong.Dong@UTDallas.edu; xdong2@jhmi.edu

This PDF file includes:

Figs. S1 to S4

Fig. S1.

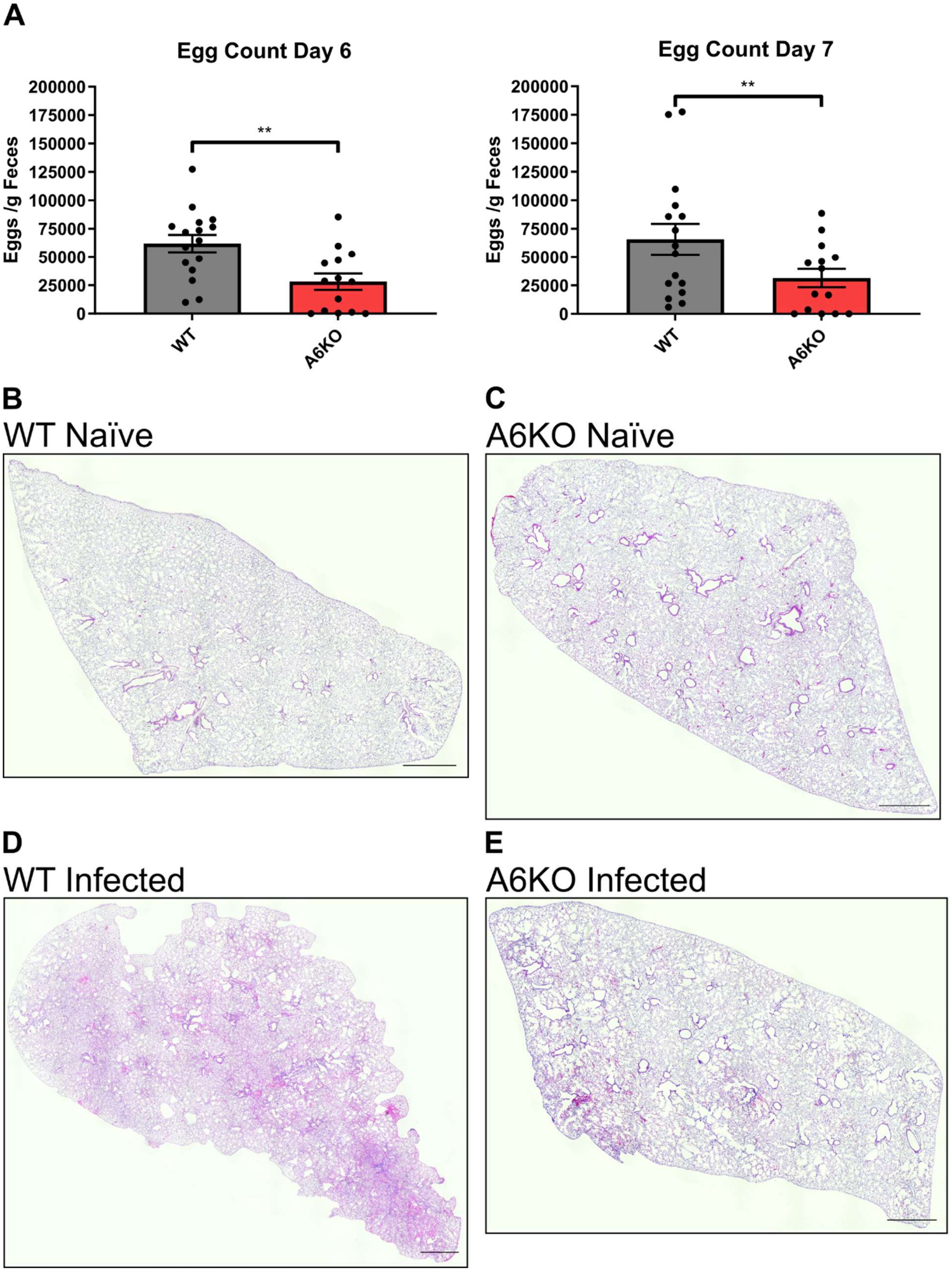

Expanded phenotypic and histological characterization of helminth infection.

(A) Day 6 and 7 post-infection egg counts (eggs per gram of feces) from WT and A6KO mice. n = 14-16
(B-E) Representative H&E-stained lung sections from WT and A6KO mice under naïve and day 3 post-infection conditions. Scale bar, 1000 μm.

Results in (A) are presented as mean ± SEM from at least three independent experiments. $^{**}p < 0.01$ (two-tailed unpaired Student's t test).

Fig. S2.

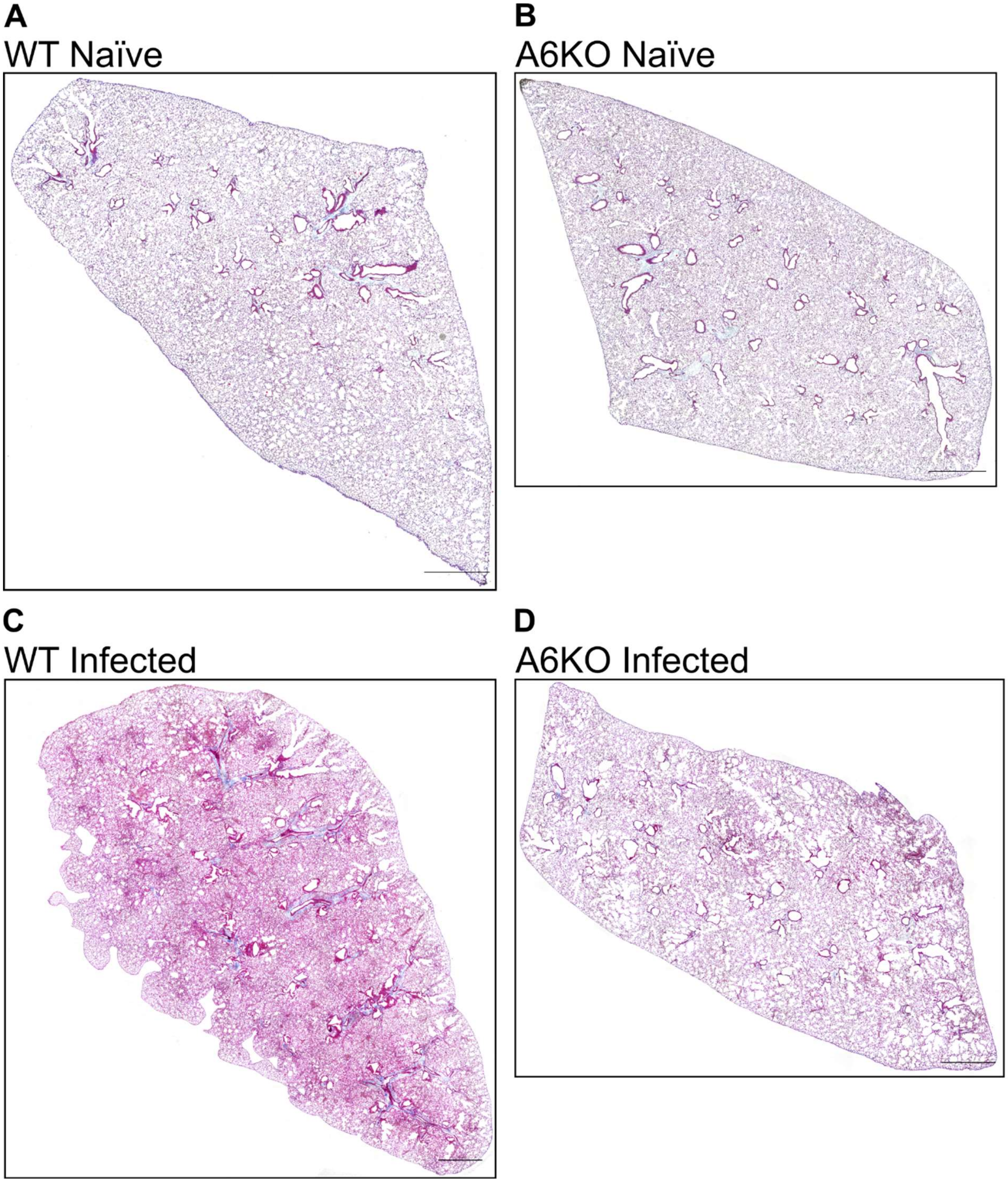

Representative Masson’s trichrome–stained lung sections during early helminth infection. (A-D) Masson’s trichrome–stained lung sections from WT and A6KO mice under naïve and day 2 post-infection conditions. Images were acquired and processed identically for brightness and contrast. Scale bar, 1000 μm.

Fig. S3.

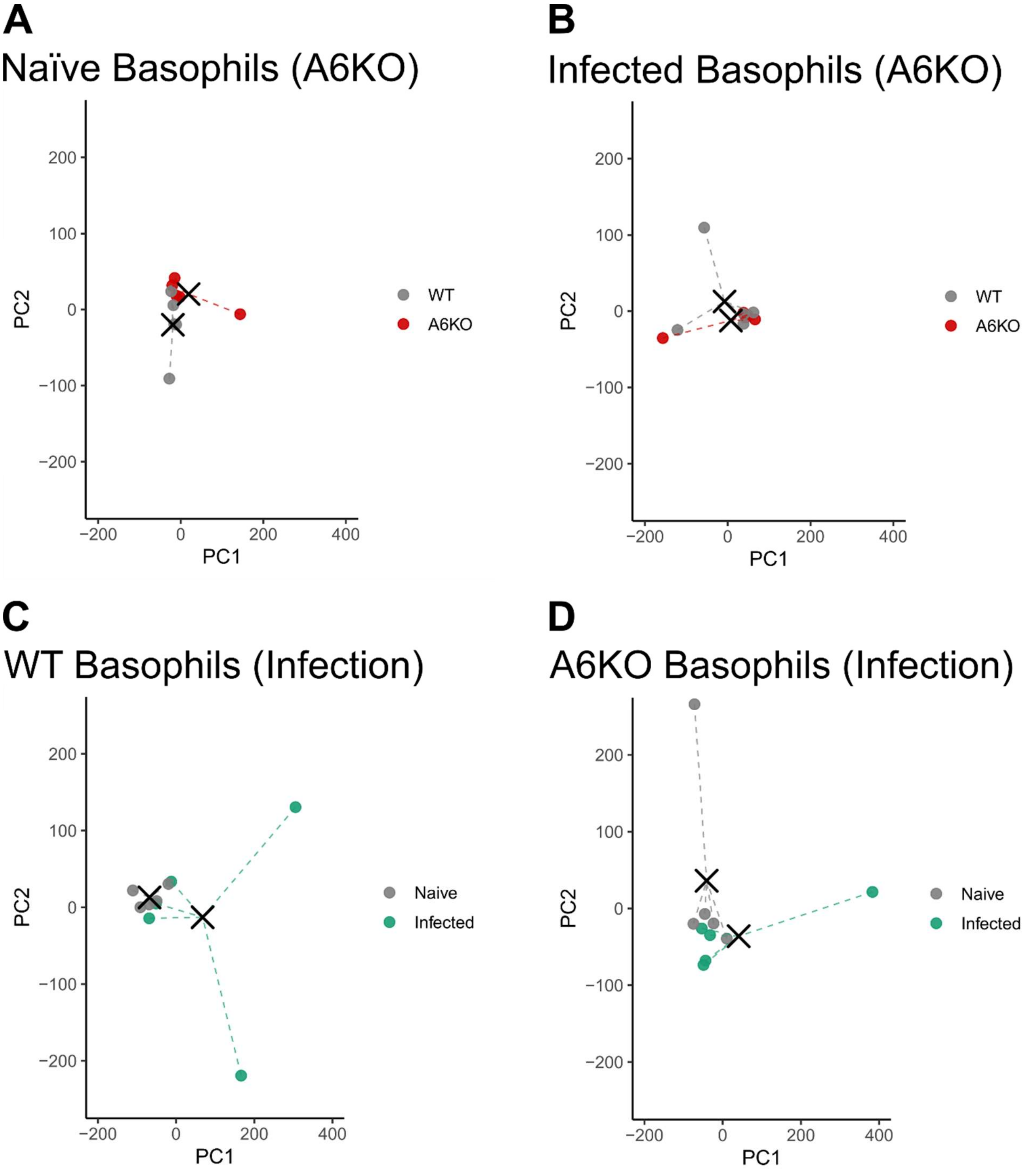

Principal component analysis of basophils during helminth infection.
(A–D) Principal component analysis (PCA) of lung basophils from WT and A6KO mice under naïve and day 3 post-infection conditions. Plots illustrate transcriptional differences associated with A6KO (A, B) or helminth infection (C, D). Black crosses indicate group centroids. Permutation testing of centroid separation revealed significant separation between naïve WT and A6KO basophils (A, $p = 0.0356$) and a trend toward separation between naïve and infected WT basophils (C, $p = 0.0751$). No significant separation was observed between infected WT and

A6KO basophils (B, $p = 0.842$) or between naïve and infected A6KO basophils (D, $p = 0.423$). n = 5 mice per group.

Fig S4.

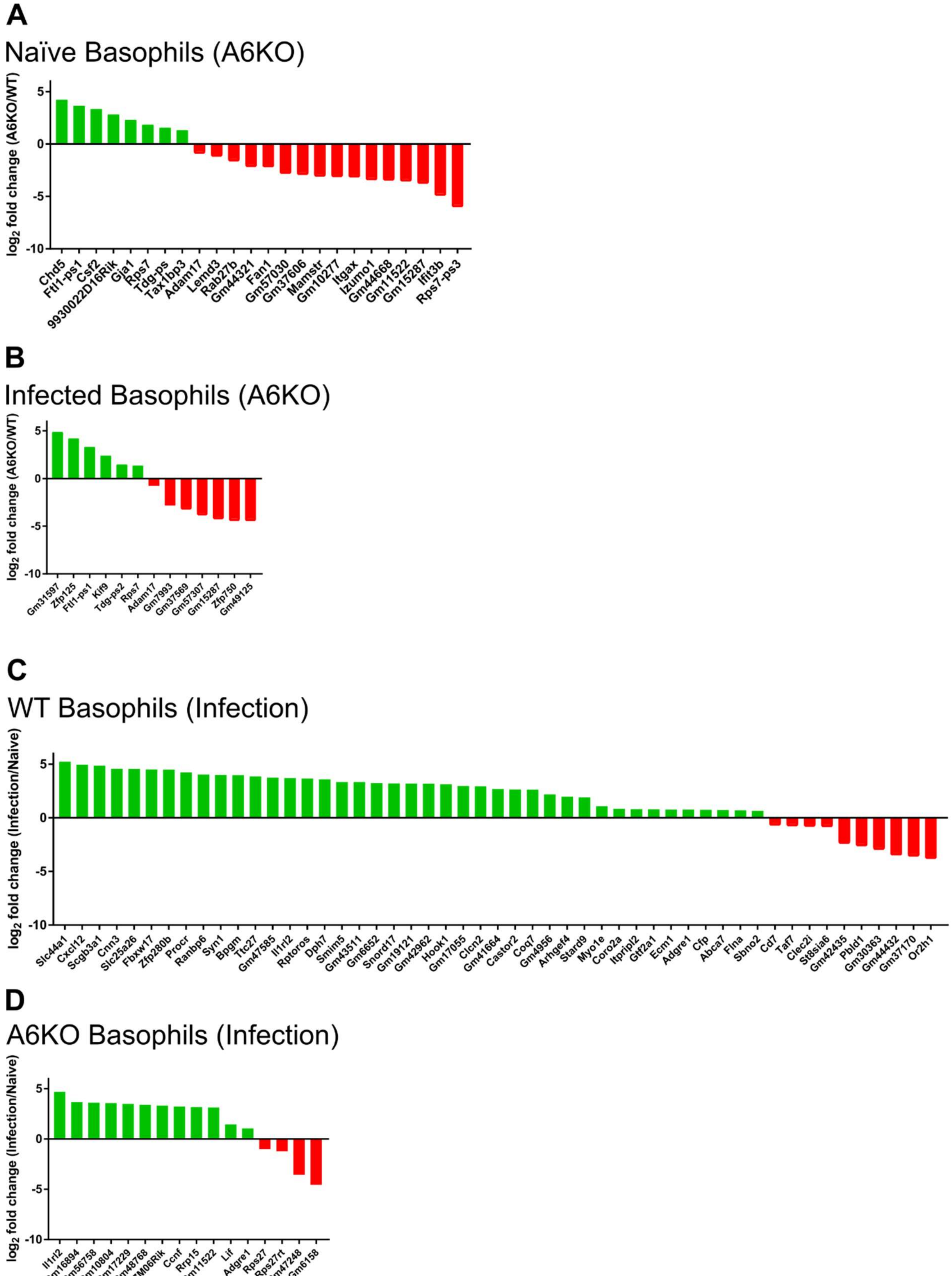
A
Naïve Basophils (A6KO)
log2 fold change (A6KO/WT)
5
0
-5
-10
Chd5
Ftl1-ps1
Csf2
9930022D16Rik
Gja1
Rps7
Tdg-ps
Tax1bp3
Adam17
Lemd3
Rab27b
Gm44321
Fan1
Gm57030
Gm37606
Mamstr
Gm10277
Itgax
Izumo1
Gm44668
Gm11522
Gm15287
Ifit3b
Rps7-ps3
B
Infected Basophils (A6KO)
log2 fold change (A6KO/WT)
Gm31597
Zfp125
Ftl1-ps1
Klf9
Tdg-ps2
Rps7
Adam17
Gm7993
Gm37569
Gm57307
Gm15287
Zfp750
Gm49125
C
WT Basophils (Infection)
log2 fold change (Infection/Naive)
Slc44a1
Cxcl12
Scgb3a1
Cnn3
Slc25a26
Fbxw17
Zfp280b
Procr
Ranbp6
Syn1
Bpgm
Ttc27
Gm47585
Il1rl2
Rptoros
Dph7
Smim5
Gm43511
Gm6652
Snord17
Gm19121
Gm42962
Hook1
Gm17055
Clcn2
Gm41664
Castor2
Coq7
Gm4956
Arhgef4
Stard9
Myo1e
Coro2a
Itpripl2
Gtf2a1
Ecm1
Adgre1
Cfp
Abca7
Flna
Sbno2
Cd7
Taf7
Clec2i
St8sia6
Gm42435
Pbld1
Gm30363
Gm44432
Gm37170
Or2h1
D
A6KO Basophils (Infection)
log2 fold change (Infection/Naive)
Il1rl2
Gm16894
Gm56758
Gm10804
Gm17229
Gm48768
6330537M06Rik
Ccnf
Rrp15
Gm11522
Lif
Adgre1
Rps27
Rps27rt
Gm47248
Gm6158

Complete list of significantly differentially expressed genes in basophils
(A-D) Bar graphs showing effect sizes (β), representing $\log_2$ fold change, for DEGs in basophils due to Mrgpra6 knockout (A6KO) or N. brasiliensis infection (Infection). Number of DEGs are 24 (A), 13 (B), 51 (C), and 16(D).

Differentially expressed genes were defined using an FDR-adjusted q-value < 0.10, n = 5 individual mice per genotype per condition (WT Naïve, A6KO Naïve, WT Infected, A6KO Infected)
